# A Novel *Alteromonas* Phage Lineage with a Broad Host Range and Small Burst Size

**DOI:** 10.1128/spectrum.01499-22

**Published:** 2022-07-11

**Authors:** Yahui Yang, Ruijie Ma, Chen Yu, Junlei Ye, Xiaowei Chen, Long Wang, Nianzhi Jiao, Rui Zhang

**Affiliations:** a State Key Laboratory of Marine Environmental Science, Fujian Key Laboratory of Marine Carbon Sequestration, College of Ocean and Earth Sciences, Xiamen University, Xiamen, China; b College of Ocean and Earth Sciences, Xiamen University, Xiamen, China; c Southern Marine Science and Engineering Guangdong Laboratory (Zhuhai), Zhuhai, China; Huazhong University of Science and Technology

**Keywords:** *Alteromonas*, burst size, host range, auxiliary metabolic genes, comparative genomic analysis

## Abstract

*Alteromonas* is an opportunistic marine bacterium that persists in the global ocean and has important ecological significance. However, current knowledge about the diversity and ecology of alterophages (phages that infect *Alteromonas*) is lacking. Here, three similar phages infecting Alteromonas macleodii ATCC 27126^T^ were isolated and physiologically characterized. Transmission electron microscopy revealed *Siphoviridae* morphology, with an oblate icosahedral head and a long noncontractile tail. Notably, these members displayed a small burst size (15–19 plaque-forming units/cell) yet an extensively broad host spectrum when tested on 175 *Alteromonas* strains. Such unique infection kinetics are potentially associated with discrepancies in codon usage bias from the host tRNA inventory. Phylogenetic analysis indicated that the three phages are closely evolutionarily related; they clustered at the species level and represent a novel genus. Three auxiliary metabolic genes with roles in nucleotide metabolism and putative biofilm dispersal were found in these phage genomes, which revealed important biogeochemical significance of these alterophages in marine ecosystems. Our isolation and characterization of these novel phages expand the current understanding of alterophage diversity, evolution, and phage–host interactions.

**IMPORTANCE** The marine bacterium *Alteromonas* is prevalent in the global ocean with crucial ecological significance; however, little is known about the diversity and evolution of its bacteriophages that profoundly affect the bacterial communities. Our study characterized a novel genus of three newly isolated *Alteromonas* phages that exhibited a distinct infection strategy of broad host spectrum and small burst size. This strategy is likely a consequence of the viral trade-off between virulence and lysis profiles during phage–host coevolution, and our work provides new insight into viral evolution and infection strategies.

## INTRODUCTION

*Alteromonas* is a copiotrophic bacterium of Gammaproteobacteria that is widely distributed in diverse marine environments worldwide, including coastal waters and open oceans, ranging from the surface to bathypelagic seawaters and deep-sea sediments (1, 2). *Alteromonas* has been considered an *r*-strategist (emphasizing reproduction over survival) because of its rapid response to transient organic nutrients during environmental perturbations such as phytoplankton bloom (3, 4). Moreover, it is adapted to living as both plankton and particle attachments including biofilms or marine snow (5, 6). Collectively, the genus *Alteromonas* is one of the most readily and frequently isolated heterotrophic bacterium, with 29 validated species published to date (https://www.bacterio.net/genus/alteromonas). In recent studies, *Alteromonas* was characterized as a siderophore producer, capable of facilitating iron acquisition from specific sources in the global marine community (7, 8). *Alteromonas* is also known as a helper bacterium of *Prochlorococcus*, a dominant marine primary producer (9), by protecting it against damage from hydrogen peroxide (10). Therefore, *Alteromonas* has received substantial attention in recent years as a model strain for marine bacterial research because of its significant ecological roles.

As the most abundant biological agents, viruses play critical roles in structuring microbial populations and communities; they affect the nutrient cycle and energy flow within microbial loops, thereby regulating global biogeochemical cycles (11). Although metagenomic approaches have revealed the vast genetic diversity of marine viruses, there is still a large number of unknown sequences (“viral dark matter”) because of the lack of viral reference genomes in the databases, especially for ubiquitous phages that infect key microbial clades across the ocean (12). Therefore, to further extend our understanding of the diversity, evolution, and ecology of both phages and their bacterial hosts, it is essential to isolate and characterize novel phages. However, only 14 *Alteromonas*-infecting phages have been reported to date (Table S1 in the supplemental material). This is considerably lagging behind the study of phages targeting other marine bacterial clades, such as cyanophages, vibriophages and roseophages.

The currently known alterophages, including seven podophages, five siphophages, one myophage, and one filamentous phage, were isolated from the Mediterranean Sea, the coastal and offshore waters of China, and the North Sea (13–21). The infection strategies and biogeographic patterns of marine phages are closely related to their host range and burst size. However, most of these alterophages lack data from intragenus host range assays, with the exception of vB_AcoS-R7M (R7M) (19) and vB_AmeP-R8W (R8W) (20), which can infect five and nine different *Alteromonas* species, respectively. It is noteworthy that R8W exhibits a wide host range of 35 *Alteromonas* strains with a strong preference for deep-sea isolates. Burst size, the number of virions released upon infected host cell lysis, is another crucial factor for phage infection strategy. In alterophages, limited data revealed that burst size greatly varies, from 60–600 plaque-forming units (PFU)/cell (13, 14). Overall, because of the insufficient alterophage isolates and limited physiological data, the infection strategies of alterophages remain poorly understood.

In this study, three isolates that infect the type strain *A. macleodii* ATCC 27126^T^ were isolated and fully characterized. Moreover, we identified two homologous metagenome-assembled alterophage-like contigs in the public database. Our experimental and *in silico* investigation on this novel phage group provides new insights into alterophage evolution and ecology, and phage–host interactions.

## RESULTS AND DISCUSSION

### R9Y-phages exhibit broad host spectrum and small burst size.

**FIG 1:**
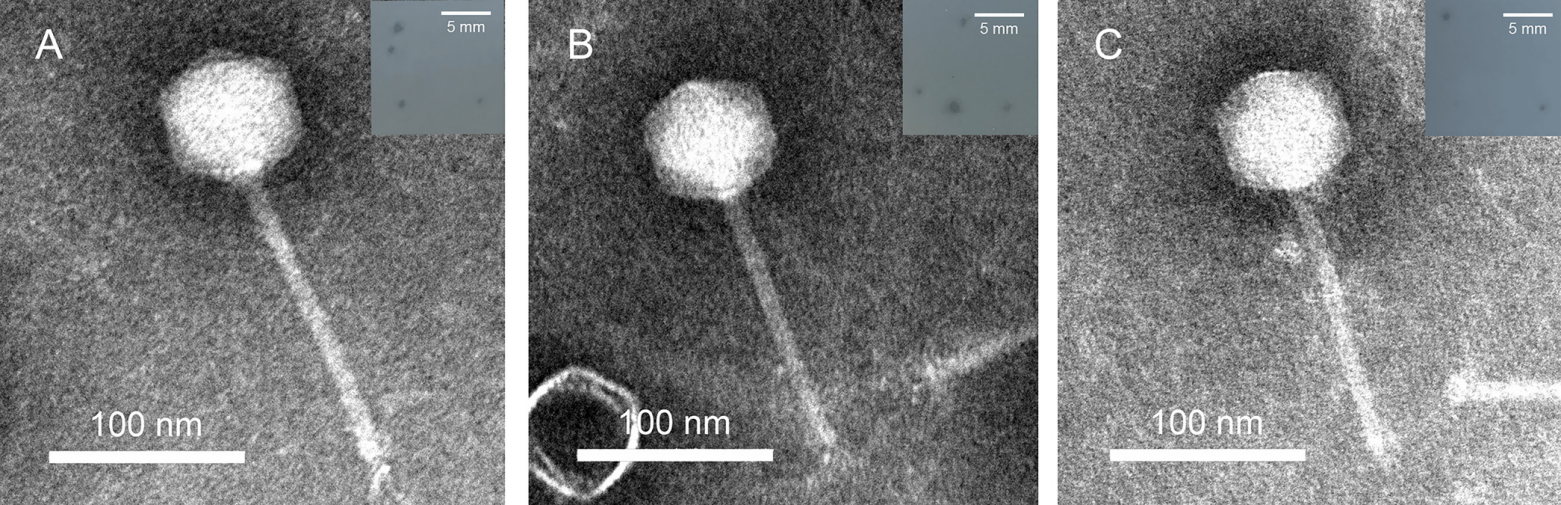
TEM images of three R9Y-alterophages. (A) vB_AmaS-R9Y1; (B) vB_AmaS-R9Y2; (C) vB_AmaS-R9Y3. Scale bar, 100 nm. Inset shows phage plaques formed on the lawn of *A. macleodii* ATCC 27126; scale bar, 5 mm.

We isolated three phages, vB_AmaS-R9Y1 (R9Y1), vB_AmaS-R9Y2 (R9Y2) and vB_AmaS-R9Y3 (R9Y3), from aquaculture water samples of three different seafood markets in Guangzhou (23.13°N, 113.30°E; 23.12°N, 113.25°E) and Zhangzhou (23.70°N, 117.42°E). Three R9Y-phages formed small round plaques (<1 mm in diameter) with a turbid halo on the host lawn (Fig. 1). Transmission electron microscopy (TEM) inspection revealed their similar morphotype to *Siphoviridae* (Fig. 1), comprising of an oblate head (68.36 ± 2.38-nm long and 64.72 ± 1.89-nm wide) and a long noncontractile tail (140.95 ± 3.86 nm). Such an oblate head is similar to that of Rhodobacter capsulatus gene transfer agent (RcGTA) (22), a tailed phage-like entity exclusively encapsulating random bacterial fragments (23).

**FIG 2:**
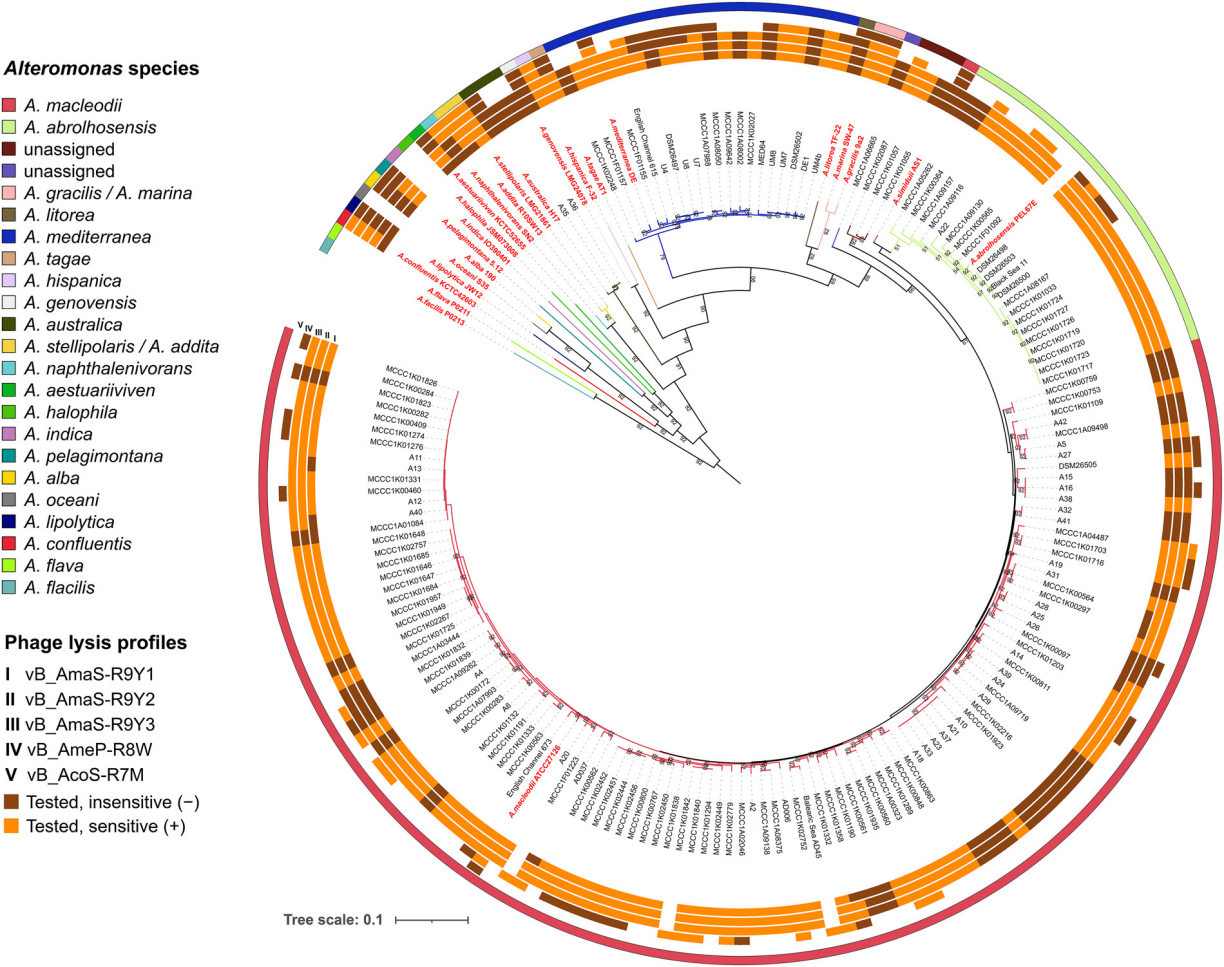
Lysis profiles of alterophages R7M(19), R8W(20), and R9Y1 to R9Y3 (this study) against 175 *Alteromonas* strains, which included 18 type strains and 157 nontype strains. Sensitive and insensitive strains are highlighted in orange and brown, respectively. Internal phylogenetic tree showing the relationships among tested bacterial strains is based on concatenated alignments of 92 core genes. Type strains of *Alteromonas* are shown in red.

To examine the host range of the three phages, a spotting test was performed against 175 *Alteromonas* strains (18 type strains of *Alteromonas* species and 157 nontype *Alteromonas* strains including 119 *A. macleodii*, 16 *Alteromonas abrolhosensis*, 18 *Alteromonas mediterranea*, 1 *Alteromonas australica*, and 3 unclassified *Alteromonas* sp. strains). The results showed that R9Y1 could lyse 8 of the 18 type strains, and R9Y2 and R9Y3 could lyse 11 of them (Fig. 2). Compared with two recently reported alterophages, R7M (19) and R8W (20), that can infect 5 and 9 different *Alteromonas* species, respectively, against the same collection of *Alteromonas* type strains, R9Y2 and R9Y3 displayed the broadest host spectrum. Moreover, among 157 nontype *Alteromonas* strains, the lethal rate of phages R9Y1, R9Y2, and R9Y3 reached 67.53%, 72.08%, and 70.13%, respectively (Fig. 2; see also Table S2). These values were significantly higher than that of another *A. macleodii* ATCC 27126-targeting phage, R8W (43.48%), against the same collection of 69 strains of various ecotypes determined by Pearson chi-square test or Fisher’s exact test (Fig. S1). Furthermore, strains that are susceptible to R9Y-phages were isolated from multiple habitats, including nearshore estuaries and global oceans (Pacific, Indian, and Atlantic Oceans) ranging from surface to bathypelagic seawaters and deep-sea sediments. Therefore, R9Y-phages may be prevalent and active killers of *Alteromonas* in the ocean, posing significant effects on the fitness and dynamics of the host population. Such lytic phages with a broad host range may also have profound ecological significance to gene transfer (24).

**FIG 3:**
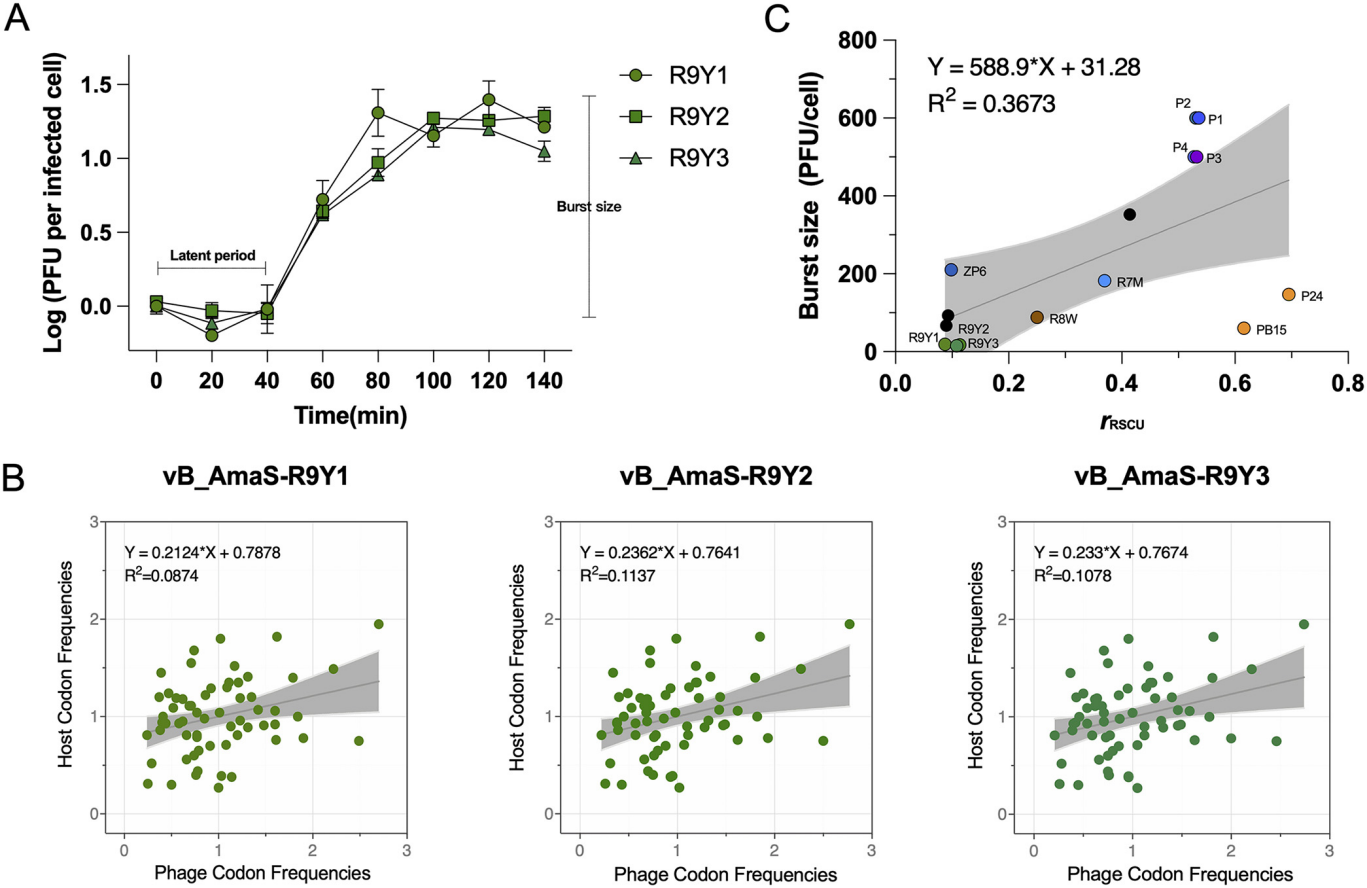
(A) One-step growth curves of R9Y1, R9Y2, and R9Y3 infecting the trapping host *A. macleodii* ATCC 27126. The data shown are average values from triplicate experiments, and the error bars represent standard deviations. (B) Correlation analysis of R9Y1, R9Y2, and R9Y3 RSCU values of 62 codons with their hosts. Gray shadows indicate the best-fit line with 95% confidence intervals from linear regression. (C) Correlation analysis of *r*_RSCU_ values and available burst size data for reported alterophages and three unpublished lytic alterophages (black circle). The gray shadow indicates the best-fit line with 95% confidence intervals from linear regression.

To further understand the infection kinetics of R9Y-phages, the one-step growth curve was examined. The results showed ta latent period of 40 min and a rapid growing period of 40–60 min, with burst sizes of 19, 17, and 15 PFU/cell for R9Y1, R9Y2, and R9Y3, respectively (Fig. 3A); these are the smallest burst sizes compared with other currently known alterosiphophages (60–182 PFU/cell) (14, 19). Furthermore, a recent study analyzed the one-step growth curves of viruses over the past 7 decades and showed that the burst sizes of viruses spanned a range from 10 to 1,000 PFU per cell (25). That is, the burst size of R9Y-phages is exceedingly close to the minimum burst size of known viruses.

Because R9Y-phages lack tRNA genes of their own, codon usage bias adaptation to their hosts is essential and expected. We calculated 62-codon relative synonymous codon usage (RSCU) values (except for two encoding Met and Trp) of each phage–host pair, and *r*_RSCU_ (correlation between host and phage RSCU values) was used to assess viral capacity to exploit host tRNA inventory during translation (26). Three R9Y-phages showed extremely small *r*_RSCU_ values (0.08–0.11) and imperfect symmetrical distribution (slope, 0.21–0.23) (Fig. 3B), which indicates poor codon adaptation to the host translation machinery and limited ability to use the host tRNA inventory. This may explain their extremely small burst size. Indeed, combined with data from the currently reported alterophages with known burst size, we observed a significant trend: lower *r*_RSCU_ is likely correlated with smaller burst size (*P = *0.0166; Fig. 3C).

These results demonstrated that R9Y-phages displayed small burst size but broad host spectrum. Such distinct infection kinetics correspond to the arms race dynamics (ARD) model of phage–host coevolution, in which the host resistance and phage lysis profile iteratively increase through time (27). Namely, phages likely evolved from specialist to generalist at the cost of fewer phage descendants production or slower replication rate, which reflects the antagonistic pleiotropy of phage mutation (28, 29). Hence, we demonstrated that this physiological trait of R9Y-phages may represent a replication strategy of alterophages in which phages likely sacrifice the chance to maximize host resources during infection in exchange for the ability to infect a wider host range.

### General genomic features and comparisons of R9Y-phages.

**FIG 4:**
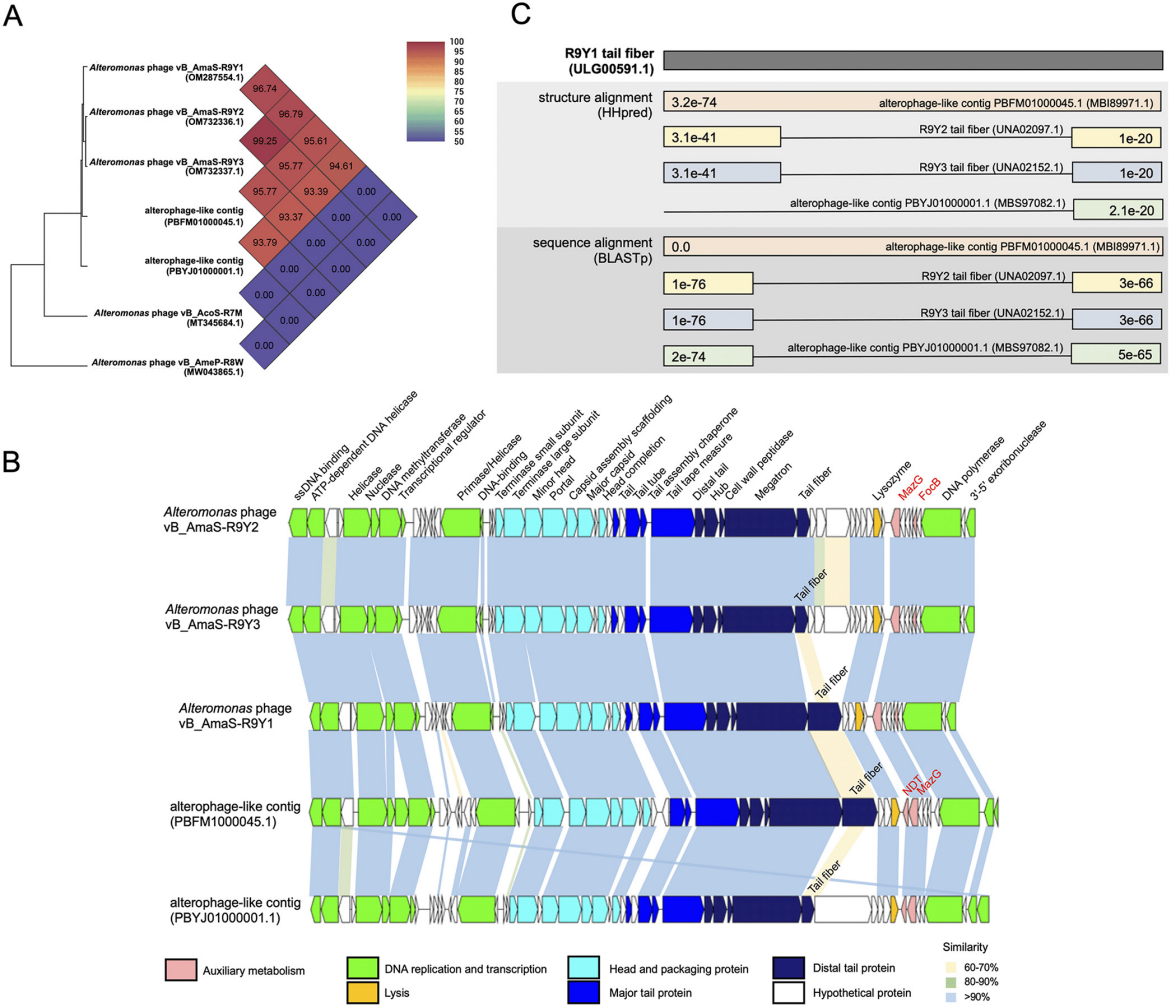
Comparative genomic analyses of R9Y-related phages. (A) Whole-genome phylogeny based on the ANI of five R9Y-related alterophages and two alterophage outgroups using BLASTn. (B) Whole-genome comparisons of five R9Y-related alterophages. Homologous ORFs are connected using shadows of different colors, which indicates different pairwise BLASTp identities. Phage ORFs are oriented according to the direction of transcription. Gene functions are colored according to categories noted below. Annotation of phage AMGs are highlighted in red. (C) Protein structure and amino acid sequence alignments of tail fibers from R9Y-related phages. E-values of HHpred and BLASTp alignments are shown.

R9Y-phages are double-stranded DNA viruses with a genome length ranging from 40 kb to 43 kb and a G+C content of 54.9% to 55.1% (Table S1). R9Y1, R9Y2, and R9Y3 were predicted to have 50, 55, and 56 open reading frames (ORFs), respectively. According to the BLASTp results, 75%–86% of ORFs were homologous to those predicted in two alterophage-like contigs (GenBank PBFM01000045.1 and PBYJ01000001.1) of metagenome-assembled genomes from the Pacific Ocean (30), and these five members shared approximately 93%–95% pairwise genome-wide average nucleotide identities (ANI) (Fig. 4A). Furthermore, the recruited metagenomic data indicated that R9Y-phages were present at four sites of the *Tara* Oceans expedition at various depths, from the surface to mesopelagic zones, in the South and North Pacific Oceans (Table S3).

In total, 35 ORFs were conserved among all members, which showed an isomorphic genomic organization (Fig. 4B). Such great overall synteny also suggested that they were derived from a common ancestor. Moreover, no lysogeny-related genes (e.g., integrase, transposase, and repressor) were identified, which indicates that these members are likely to be obligate lytic.

A major difference between the five members is the tail fiber gene; some members shared a pairwise identity as low as 64%. Previous studies have also shown that baseplate-related tail genes, especially the tail fiber gene, likely undergo more frequent horizontal transfers for viral host jumps (19, 31). However, when aligned using HHpred and BLASTp, both termini of the five phages were found to match to each other well, both genetically and structurally (Fig. 4C). The conserved N terminus ensures correct attachment to the megatron fiber-binding domain, whereas the highly similar C termini are putative receptor-binding domains specific to *Alteromonas* (19). Although we found subtle differences in the lysis profiles among three cultivable alterophages, it is unclear whether these are caused by the difference in the middle segment of the tail fiber gene. Moreover, the contents of their auxiliary metabolic genes (AMGs) also slightly differed, which are described in the AMGs identification section.

### Phylogenetic and taxonomic analysis reveal R9Y-related phage novelty.

**FIG 5:**
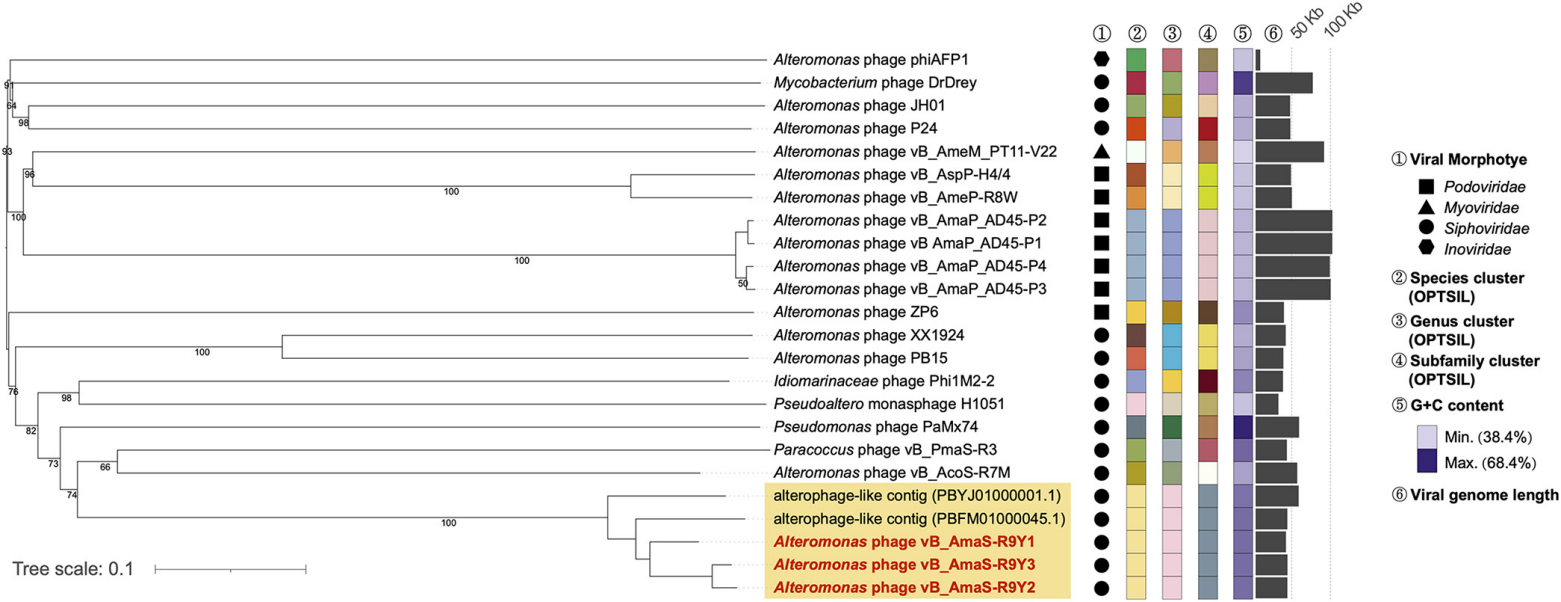
Taxonomy of R9Y-phages. The GBDP tree based on the complete amino acid profiles of phages. Pseudobootstrap support values greater than 50/100 replications are shown.

To explore the taxonomy of R9Y-related phages, a marker-gene survey using genes encoding DNA polymerase and major capsid protein was conducted. Both phylogenetic trees showed that these five R9Y-related phages established distinct clustering (Fig. S2). A phylogeny of four concatenated RcGTA-like baseplate-related genes (e.g., ORF15–ORF18 in R9Y1), which were found mostly preserved in numerous marine bacterial and viral genomes (32), was also constructed. Likewise, these five phages again formed a tightly cohesive cluster (Fig. S3). More importantly, a Genome-BLAST Distance Phylogeny (GBDP) tree (Fig. 5) based on complete phage amino acid profiles also demonstrated that these R9Y-related phages are closely evolutionarily associated and remarkably novel.

The five related phages share pairwise ANIs of 93.37%–99.25% (Fig. 4A), which indicates that they should be categorized into a single viral species according to the criterion for classification at the viral species rank (~95% ANI [33]). Furthermore, because no classified virus shared >50% ANI with these members, this viral species should be further assigned to a novel genus (33). The assignment was also verified by OPTSIL (34) (Fig. 5), a program commonly used for virus classification.

Based on these results, we propose that R9Y-phages represent a new genus of the *Siphoviridae* family. Members of this candidate genus were either isolated from coastal aquatic sewage or recruited from the metagenomic data sampled from the Pacific Ocean, and they can infect hosts isolated from various marine habitats. Therefore, we speculate that these members likely have high adaptability to different ecological habitats.

### Identification of AMGs related to nucleotide metabolism and putative host biofilm dispersal.

AMGs are recognized as entrenched parts of viral genomes that regulate host metabolism or stress response during infection (35–37). Therefore, analysis of viral AMGs could provide further understanding of how viruses potentially affect host metabolism and further contribute to the microbial community dynamics. At the space between the viral lysis-related module and the DNA replication and transcription module, three AMGs were found among the five members. All of them shared a nucleoside triphosphate pyrophosphohydrolase-like domain (MazG). MazG was initially proposed to optimize virus infection efficiency as a modulator of the stringent host nutrient-deficient response (38, 39). Nevertheless, as MazG has been increasingly found in viruses that infect bacteria isolated from diverse eutrophic environments (19, 40), viral MazG was instead suggested to preferentially hydrolyze dGTP and dCTP; this allows the AT-rich viruses to recycle and use deoxyribonucleotides from hosts with high GC contents (41).

The AMG unique to the three cultivable isolates is a gene that encodes an adhesin biosynthesis transcription regulatory protein, the formate channel FocB. FocB is a negative regulator of the Escherichia coli *fim* operon encoding type 1 fimbria (42), an essential adhesive organelle for bacterial biofilm formation (43, 44). Thus, FocB expression likely negatively regulates biofilm formation of *Alteromonas* hosts to facilitate viral infection and transmission.

Another AMG unique to both of the nonculturable alterophages encodes a purine nucleoside 2-deoxyribosyltransferase (NDT), which plays an important role in the nucleoside salvage pathway for DNA synthesis. By catalyzing the transfer of the 2′-deoxyribose moiety between purine bases (45–47), the viral purine NDT may allow the viruses to recycle host purine nucleosides. Therefore, both nucleotide metabolism-related AMGs (*MazG* and the NDT gene) likely help the phage hijack host nucleotide biosynthesis to enhance the viral biosynthetic capacity.

Notably, all detected AMGs were located in the space between viral functional modules, which indicates that they were acquired through horizontal gene transfers (48). Moreover, *mazG* existed in all five phages, whereas *focB* and the NDT gene showed only a limited distribution; therefore, *mazG* may have been introduced by their common ancestor, whereas *focB* and the NDT gene were obtained after differentiation began.

### Conclusions.

This research elucidated the physiological and genomic characteristics of three similar *Alteromonas* phages, R9Y1, R9Y2, and R9Y3. These three phages exhibited a unique infection strategy of small burst size (15–19 PFU/cell) but broad host spectrum (lethal rates of 65%–71% against 175 *Alteromonas* strains), which was first observed in alterophages. This strategy reflects the antagonistic pleiotropy between host generalism and specialism associated with their generalized codon usage bias from broadly adapted hosts’ tRNA inventory. Together with two similar metagenome-assembled alterophage-like contigs, these five representatives of a single species can further be assigned into a novel genus of the family *Siphoviridae*. Three AMGs (*focB*, *MazG*, and the NDT gene) putatively involved in host biofilm dispersal and nucleotide metabolism were identified in this new phage group. Our comprehensive study significantly extends our knowledge of the diversity of alterophages and discloses unique infection kinetics in alterophages. Further research regarding the effect of the environmental factors, such as host density and nutrient conditions, on obscure viral infection strategies and phage–host coevolutionary patterns will allow better insights into the evolution and ecology of *Alteromonas*.

## MATERIALS AND METHODS

### Isolation and purification of phages.

Alteromonas macleodii ATCC 27126^T^, the trapping host in this study, was incubated in rich organic (RO) medium (peptone, 1 g; yeast extract, 1 g; sodium acetate, 1 g; artificial seawater, 1 L; pH 7.4–7.8) at 28°C with a shaking speed of 180 rpm/min. Then, 1 mL of each water sample from coastal waters (Xiamen coast, China), marginal seas (South China Sea), and different seafood markets (Guangzhou, Xiamen, and Zhangzhou, China) was incubated with 20 mL of an exponentially growing culture of *A. macleodii* ATCC 27126 for 24 h for enrichment. Phage lysate was then filtered through a 0.22-μm pore size syringe filter (Millipore, CA, USA) to collect the infectious virions. Subsequently, the filtered lysate was diluted and mixed with 1 mL exponentially growing host culture and 6 mL molten RO agar medium (0.5% wt/vol agar) for double-layer agar plating. After incubating overnight, a separated clear plaque was selected and further purified five times, and stored in 1 mL of storage media buffer (50 mM Tris-HCl, 0.1 M NaCl, 8 mM MgSO_4_, pH 7.5) at 4°C for later use.

To obtain high phage biomass, 1 L phage lysate was treated with 2 mg/L of DNase and RNase for 1 h at room temperature. Then, 1 M NaCl was also added, and the mixture was placed in an ice bath for 30 min to separate the phage particles from host cell debris. The mixture was then centrifuged at 10,000 × *g* for 10 min at 4°C, and the supernatant was filtered through 0.22-μm membranes, treated with polyethylene glycol 8000 (10% [wt/vol]), and stored at 4°C overnight. After centrifugation at 10,000 × *g* for 60 min at 4°C, the phage precipitate was resuspended in 6 mL of storage media buffer, which was further purified by CsCl equilibrium gradient centrifugation (200,000 × *g*, 4°C, 24 h) using an Optima L-100 XP ultracentrifuge (Beckman Coulter, CA, USA). The visible viral band was extracted and further desalted through 30-kDa super-filters (Millipore, CA, USA).

### Transmission electron microscopy.

The purified and desalted phage suspension was deposited onto copper grids with carbon-coated Formvar film for 30 min in the dark, stained with 1% phosphotungstic acid for 1 min, and further air dried. The sample was observed using a JEM-2100 transmission electron microscope (JEOL, Tokyo, Japan) at an acceleration voltage of 80 kV. Images were collected using the CCD image transmission system (Gatan Inc., CA, USA). Each phage particle was measured using ImageJ for at least five virions (49).

### Host range.

Lysis profiles were determined by spotting dilutions onto bacterial lawns of 175 *Alteromonas* strains, including 18 type strains of *Alteromonas* species, 119 *A. macleodii*, 16 *A. abrolhosensis*, 18 *A. mediterranea*, 1 *A. australica*, and 3 unclassified *Alteromonas* sp. strains (Table S2). All type strains of the *Alteromonas* genus were obtained from global biological resource centers. The isolates prefixed with “MCCC,” “DSM,” and “A” were obtained from the Marine Culture Collection of China (MCCC), the Deutsche Sammlung von Mikroorganismen und Zellkulturen (DSMZ), and the Center for Collection of Marine Bacteria (CCMB), respectively. Then, 1 mL of exponential growing host culture was mixed with 6 mL molten RO agar medium (0.5% wt/vol agar). The mixture was then poured onto a solidified RO agar plate (1.5% wt/vol agar), which was left standing at room temperature in the dark for 20 min to solidify. The phages were previously 1:100 serial diluted and then spotted onto the surface of each plate, incubated at 37°C overnight, and then checked for the presence of clear plaques.

### One-step growth curve.

To analyze the life cycle and infection kinetics of phages, a one-step growth curve was examined. The phage was added to 1 mL exponential growing culture of *A. macleodii* ATCC 27126 at a multiplicity of infection of 0.01 and then incubated for 5 min at room temperature in the dark. To remove unadsorbed phage particles, the culture was centrifuged and resuspended in 50 mL RO medium. The suspension was incubated at 28°C with continuous shaking at a speed of 180 rpm/min. Viral abundance was quantified with a double-layer agar assay every 20 min. Burst size was calculated as the ratio of the number of released phage progeny at the plateau to the initial number of host cells at the beginning of the latent period.

### DNA extraction.

The high-titer phage concentrate was treated with proteinase K (20 mg/mL), SDS (10% wt/vol), and EDTA (0.5 M), and incubated at 55°C in water for 3 h. The digested sample was then added to an equal volume of phenol:chloroform:isoamyl alcohol (25:24:1) and centrifuged at 12,000 × *g* and 4°C for 5 min. This step was repeated twice. The supernatant was sequentially purified by adding chloroform:isoamyl alcohol (24:1) and centrifuged at 12,000 × *g* and 4°C for 10 min. Then, the supernatant was mixed with isoamyl alcohol and kept at −20°C overnight. The precipitate was allowed to air dry after slowly flushing with cold 70% ethanol. Samples were resuspended in 100 μL TE buffer (10 mM Tris-HCl, 1 mM EDTA, pH 8.0) and stored at 4°C until analysis.

### Genome sequencing and bioinformatic analysis.

The genomic DNA was sequenced on the Illumina Novaseq platform with 300-bp paired-end reads. The sequencing reads were assembled *de novo* with Velvet v1.2.03 (50).

The GeneMarkS online server (51) was used to identify putative ORFs of the genome. The predicted ORFs were annotated by BLASTp search against the National Center for Biotechnology Information (NCBI) nonredundant database with an e-value of 10^−3^. CD-search (52) was used to inspect protein conserved domains, and Virfam (53) was applied to confirm our genome-based structural gene predictions. The tRNAscan-SE program was used to identify tRNA genes (54). The genomic comparison was performed using all-to-all BLASTp, and genome maps were created using a custom Java script.

### Phylogenetic analyses.

Reference genomes of the *Alteromonas* isolates for host range assay were collected from the NCBI RefSeq database (by 5 September 2018) or obtained by sequencing. The Up-to-Date Bacterial Core Gene set (UBGC) (55) pipeline was used to identify the 92 bacterial single-copy core genes found in most of the known bacterial genomes. The maximum-likelihood phylogenetic tree was constructed based on the concatenated nucleotide alignment in UBGC with 100 bootstrap iterations. The demarcation of bacterial species was based on a threshold of 95% average nucleotide identity (ANI) (56).

Two viral hallmarks (DNA polymerase and major capsid protein) (57) and four concatenated RcGTA-like baseplate-related genes were used for phylogenetic analysis. The maximum-likelihood mode with 1,000 bootstrap replicates was employed with auto-assigned best-fit models in IQ-TREE v1.6.12 (58). To explore phage taxonomic status, complete amino acid sequences of all known alterophages and relevant phages determined by PHAST (59) and BLASTp were submitted to the VICTOR server (https://ggdc.dsmz.de/victor.php) to build the whole-genome tree using the GBDP method under settings recommended for prokaryotic viruses (60). Taxon boundaries at the species, genus, and subfamily levels were evaluated using the OPTSIL program with the recommended clustering thresholds (34, 60, 61).

All phylogenetic trees mentioned above were manipulated and visualized with iTOL v5 (62). The phylogenetic tree based on ANI values was analyzed using OrthoANI (63).

### Biogeographic distribution analysis.

In total, 110 unassembled metagenomic data sets of the *Tara* Oceans project (64) were downloaded from the European Nucleotide Archive (https://www.ebi.ac.uk/ena) to evaluate the biogeographic distribution of viruses (Data Set S1). BBMap v38.90 (65) was used to map the reads from the unassembled viromes. It is generally thought that the presence of a phage in a particular sample can be determined by detecting a threshold of ≥75% genome coverage with at least 90% identity for read mapping (66). The relative abundance of viruses was calculated as reads per kilobase per million mapped reads (RPKM).

### Codon usage analysis.

The RSCU value for a codon refers to its observed frequency divided by the expected frequency under the assumption of equal usage of the synonymous codons for an amino acid (67). The RSCU values of phages and their hosts were calculated using codonW v1.4.2 (https://sourceforge.net/projects/codonw) for every amino acid. Single-codon families, such as Met coded by TGG and Trp coded by TGG, were excluded from the analysis because their RSCU value is always equal to 1 regardless of codon usage. GraphPad Prism v7 (GraphPad, CA, USA) was used to perform correlation analysis and linear regression between host and phage RSCU values.

### Data availability.

The complete genome sequences of R9Y1, R9Y2, and R9Y3 were submitted to the GenBank database under the following accession numbers: OM287554.1, OM732336.1, and OM732337.1, respectively.
