# Supplementary material for: A Novel Alteromonas Phage Lineage with a Broad Host Range and Small Burst Size
Source: Microbiol Spectr. 2022 Jul 11;10(4):e01499-22. doi: 10.1128/spectrum.01499-22 (PMC9430817; doi:10.1128/spectrum.01499-22)
Supplement: Supplemental file 1 — Supplemental material. Download spectrum.01499-22-s0001.pdf, PDF file, 3.2 MB [file spectrum.01499-22-s0001.pdf]

Supplementary Materials

Novel siphophages infecting the marine bacterium *Alteromonas* represent a distinct genus with a broad host range and small burst size

Yahui Yang[a,†], Ruijie Ma[a,†], Chen Yu[a], Junlei Ye[b], Xiaowei Chen[a], Long Wang[a,c], Nianzhi Jiao[a], Rui Zhang[a,c,#]

[a]State Key Laboratory of Marine Environmental Science, Fujian Key Laboratory of Marine Carbon Sequestration, College of Ocean and Earth Sciences, Xiamen University, Xiamen, 361102, China

[b]College of Ocean and Earth Sciences, Xiamen University, Xiamen, 361102, China

[c]Southern Marine Science and Engineering Guangdong Laboratory (Zhuhai), Zhuhai, 519080, China

[#]Address correspondence to Rui Zhang, ruizhang@xmu.edu.cn.

[†]These authors contributed equally to this work.

Contents

Supplementary Figure S1. The lethal rates of R9Y1, R9Y2, R9Y3, and R8W against 69 hosts.

Supplementary Figure S2. Maximum-likelihood phylogenetic trees based on amino acid sequences of phage DNA polymerase and major capsid proteins.

Supplementary Figure S3. Maximum-likelihood phylogenetic tree based on concatenated amino acid sequences of four RcGTA-like gene.

Supplementary Table S1. General features of currently known alterophages.

Supplementary Table S2. Host range of R9Y1, R9Y2, R9Y3, and R8W against 175 tested strains of *Alteromonas*.

Supplementary Table S3. Abundance and location information of R9Y-phages found through read mapping.

Supplementary Dataset S1. *Tara* Oceans samples description.

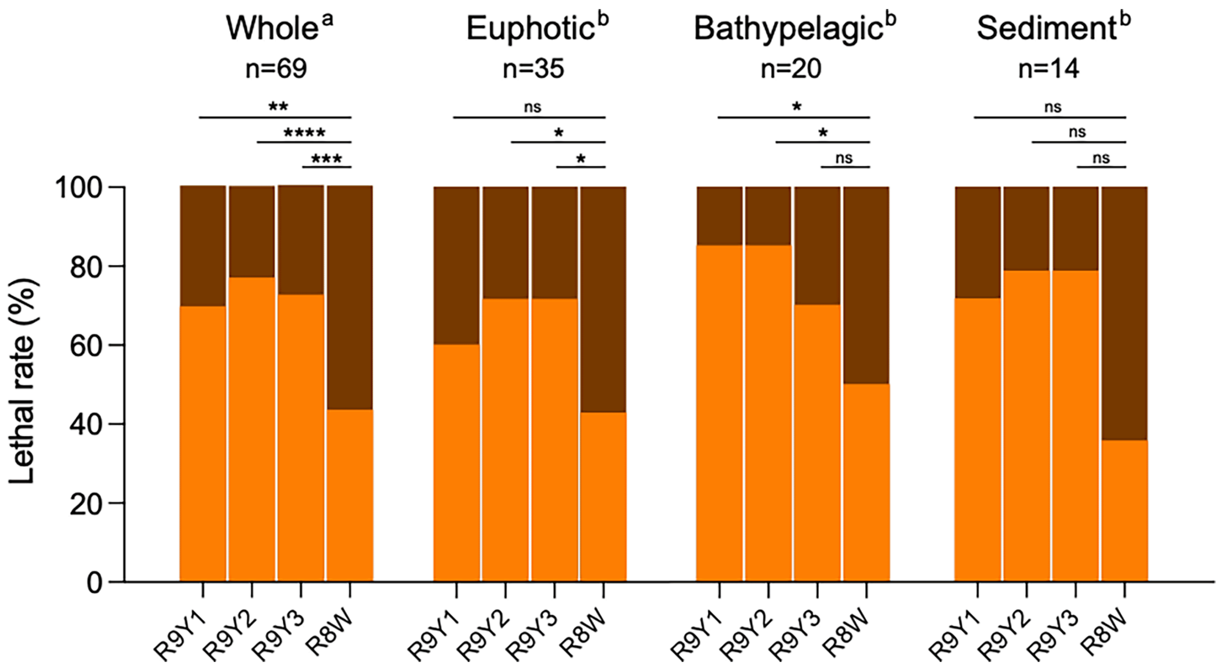

Supplementary Figure S1. The lethal rates of R9Y1, R9Y2, R9Y3, and R8W(1) against 69 hosts of three marine environmental sources. Asterisks indicate the results of [a]Pearson chi-square test or [b]Fisher’ s exact test using GraphPad Prism v7 (GraphPad, CA, USA); *p<0.05; **p<0.01; ***p<0.001; ****p<0.0001.

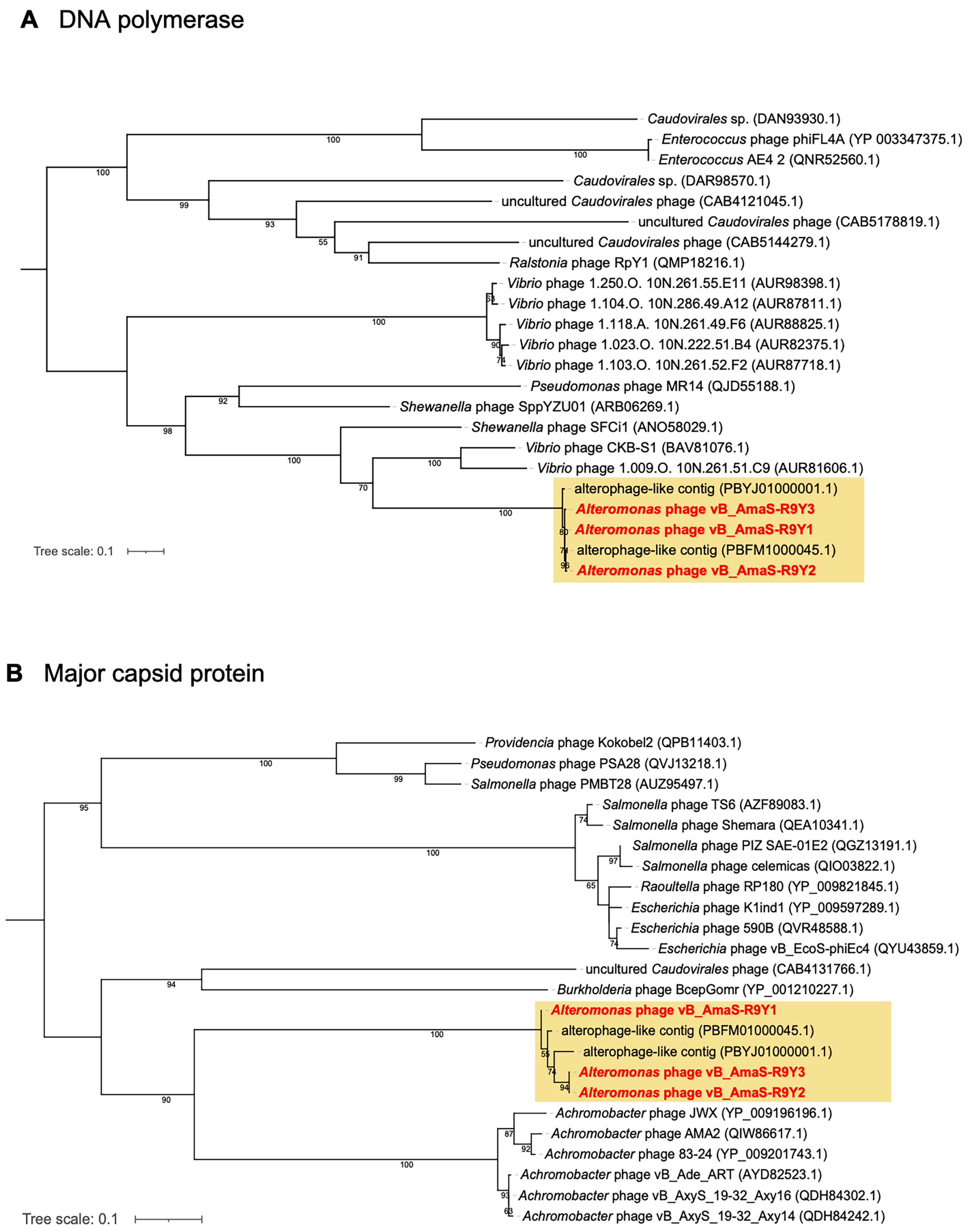

Supplementary Figure S2. Maximum-likelihood phylogenetic trees based on amino acid sequences of phage DNA polymerase (A) and major capsid proteins (B). Bootstrap values are based on 1,000 replicates.

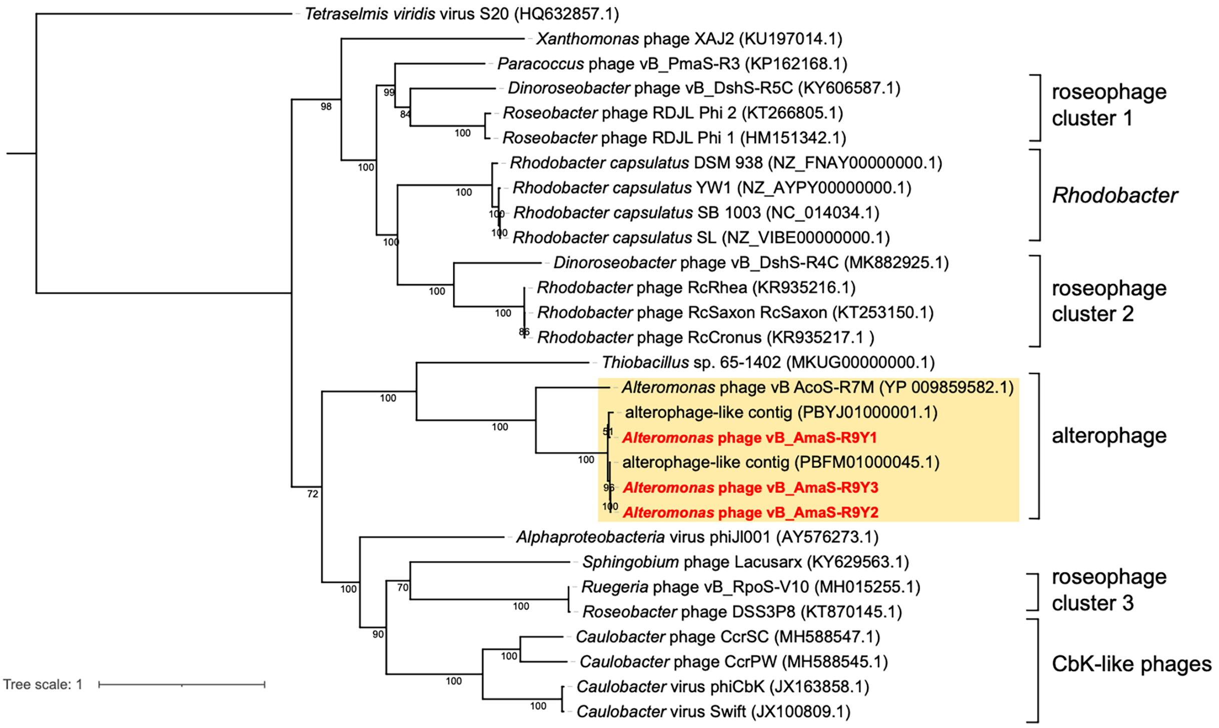

Supplementary Figure S3. Maximum-likelihood phylogenetic tree based on concatenated amino acid sequences of four RcGTA-like genes from R9Y-phages and other known phages or bacteria. Bootstrap percentage analyses were based on 1,000 replicates. R9Y-phages are marked in bold and red. The five members of our proposed genus are highlighted in orange.

Supplementary Table S1. General features of currently known alterophages.

phage	Host	Origin	Morphology	Length (bp)	G+C (%)	GenBank accession no.	Reference
AltAD45-P1	*A. macleodii* AD45	a fish farm in the Mediterranean Sea	*Podoviridae*	103,910	43.2	KF005317.1	(2)
AltAD45-P2	*A. macleodii* AD45	a fish farm in the Mediterranean Sea	*Podoviridae*	104,036	43.2	KF005320.1	(2)
AltAD45-P3	*A. macleodii* AD45	a fish farm in the Mediterranean Sea	*Podoviridae*	101,724	43.2	KF005318.1	(2)
AltAD45-P4	*A. macleodii* AD45	a fish farm in the Mediterranean Sea	*Podoviridae*	100,619	43.2	KF005319.1	(2)
PB15	*Alteromonas gracilis* B15	the Yellow Sea, China	*Siphoviridae*	37,333	45.5	KX982260.1	(3)
vB_AspP-H4/4	*Alteromonas addita* H4	North Sea water	*Podoviridae*	47,631	40.8	MF278336.1	(4)
JH01	*Alteromonas marina* SW-47^T	Qingdao coast, China	*Siphoviridae*	46,500	44.4	MH445500.1	(5)
vB_AmeM_PT11-V22	*Alteromonas mediterranea* PT11	western Mediterranean coastal waters, Spain	*Myoviridae*	92,760	38.4	MN877442.1	(6)
P24	*A. macleodii*	Qingdao coast, China	*Siphoviridae*	33,567	43.7	MK241539.2	(7)

Supplementary Table S1. *Cont).*

phage	Host	Origin	Morphology	Length (bp)	G+C (%)	GenBank accession no.	Reference
vB_AcoS-R7M	*Alteromonas confluentis* DSSK2-12^T	Xiamen coast, China	*Siphoviridae*	56,163	45.6	MT345684.1	(8)
vB_AmeP-R8W	*A. mediterranea* DE^T	Xiamen coast, China	*Podoviridae*	48,825	40.6	MW043865.1	(1)
ZP6	*A. macleodii*	Qingdao coast, China	*Podoviridae*	37,743	50.1	MK203850.1	(9)
XX1924	*Alteromonas litorea* TF-22^T	Yellow Sea, China	*Siphoviridae*	40,580	43.7	MN592896.1	unpublished
phiAFP1	*A. macleodii*	South China Sea, China	*Inoviridae*	5,859	40.6	MT975991.1	unpublished
vB_AmaS-R9Y1	*A. macleodii* ATCC 27126^T	a seafood market in Guangzhou, China	Siphoviridae	40,851	55.1	OM287554.1	This study
vB_AmaS-R9Y2	*A. macleodii* ATCC 27126^T	a seafood market in Zhangzhou, China	Siphoviridae	42,815	55.0	OM732336.1	This study
vB_AmaS-R9Y3	*A. macleodii* ATCC 27126^T	a seafood market in Guangzhou, China	*Siphoviridae*	43,008	54.9	OM732337.1	This study
alterophage-like contig	NA	Pacific Ocean	*Siphoviridae**	42,718	54.0	PBFM01000045.1	This study
alterophage-like contig	NA	Pacific Ocean	*Siphoviridae**	58,202	54.7	PBYJ01000001.1	This study

^{T}The type strain of the *Alteromonas* species.

NA indicates that the information is not available.

*The morphology of two alterophage-like contigs was predicted by Virfam(10).

Supplementary Table S2. Host range of vB_AmaS-R9Y1, R9Y2, R9Y3, and vB_AmeP-R8W against 175 tested strains of *Alteromonas*.

Strain	Taxonomy	Origin	Depth	R9Y1	R9Y2	R9Y3	R8W[a]	R7M[b]
LMG 21861[T]	*A. stellipolaris*	Antarctica	Surface	+	+	+	+	+
F-32 [T]	*A. hispanica*	Fuente de Piedra, southern Spain	Hypersaline water	+	+	+	+	+
ATCC 27126 [T]	*A. macleodii*	Hawaii, Pacific Ocean Oahu	Surface	+	+	+	+	−
9a2 [T]	*A. gracilis*	Pacific Ocean	Sediment (6310 m)	+	+	+	+	−
DE [T]	*A. mediterranea*	Adriatic Sea, Urania Basin	Bathypelagic (1000 m)	+	+	+	+	−
SW-47 [T]	*A. marina*	Eastern Sea, Korea	Surface	+	+	+	+	−
R10SW13 [T]	*A. addita*	Chazhma Bay, Sea of Japan, Pacific Ocean	Surface	−	+	+	+	+
KCTC42603 [T]	*A. confluentis*	Jeju Island, South Korea	Surface	−	+	+	+	+
AT1 [T]	*A. tagae*	Er-Jen River estuary, Tainan	Surface estuarine	+	+	+	−	−
5.12 [T]	*A. pelagimontana*	Indian Ocean	Sediment (2681 m)	+	+	+	−	−
KCTC52655 [T]	*A. aestuariivivens*	Tropical Pacific Ocean	Sediment (tidal-flat)	−	+	+	−	−
SN2 [T]	*A. naphthalenivorans*	Southern Atlantic Ocean	Sediment (tidal-flat)	−	−	−	+	+
TF-22 [T]	*A. litorea*	Korea, Yellow Sea	Sediment (Intertidal)	−	−	−	−	−
AS1 [T]	*A. simiduii*	Er-Jen River estuary, Tainan	Surface estuarine	−	−	−	−	−
JW12 [T]	*A. lipolytica*	Arabian Sea, Indian Ocean	Surface	−	−	−	−	−
H 17 [T]	*A. australica*	Port Phillip Bay, Tasman Sea, Pacific Ocean	Surface	−	−	−	−	−
190 [T]	*A. alba*	Western Pacific Ocean	Surface	−	−	−	−	−
LMG 24078 [T]	*A. genovensis*	Genoa, Italy	Biofilm	−	−	−	−	−
DSM26503	*A. macleodii*	Black Sea Karadag	Surface	+	+	+	+	NA

Supplementary Table S2. *Cont)*

Strain	Taxonomy	Origin	Depth	R9Y1	R9Y2	R9Y3	R8W[a]	R7M[b]
AD006	*A. macleodii*	Port Dickson, Malaysia	Surface	+	+	+	+	NA
MCCC 1K00172	*A. macleodii*	South China Sea	Surface	+	+	+	+	NA
MCCC 1K00560	*A. macleodii*	Eastern Pacific Ocean	Surface	+	+	+	+	NA
MCCC 1K01332	*A. macleodii*	East Pacific Ocean	Surface	+	+	+	+	NA
MCCC 1K01840	*A. macleodii*	Western Pacific Ocean	Subsurface (30 m)	+	+	+	+	NA
MCCC 1K01294	*A. macleodii*	Western Pacific Ocean	Subsurface (75 m)	+	+	+	+	NA
MCCC 1A04487	*A. macleodii*	Northwestern Pacific Ocean	Bathypelagic (2700 m)	+	+	+	+	NA
MCCC 1A07993	*A. macleodii*	Southern Atlantic Ocean	Bathypelagic (2147 m)	+	+	+	+	NA
MCCC 1A09262	*A. macleodii*	Southern Atlantic Ocean	Bathypelagic (3047 m)	+	+	+	+	NA
MCCC 1A00323	*A. macleodii*	Atlantic Ocean	Bathypelagic (3542 m)	+	+	+	+	NA
MCCC 1K02779	*A. macleodii*	Atlantic Ocean	Sediment (2577 m)	+	+	+	+	NA
BS11	*A. macleodii*	Black Sea Karadag	Surface	+	+	+	−	NA
DSM26498	*A. macleodii*	Black Sea Karadag	Surface	+	+	+	−	NA
EC673	*A. macleodii*	English Channel	Surface	+	+	+	−	NA
DSM26500	*A. macleodii*	Black Sea Karadag	Surface	+	+	+	−	NA
MCCC 1K00460	*A. macleodii*	Western Pacific Ocean	Surface	+	+	+	−	NA
MCCC 1K00811	*A. macleodii*	South China Sea	Surface	+	+	+	−	NA
MCCC 1K01274	*A. macleodii*	Western Pacific Ocean	Subsurface (100 m)	+	+	+	−	NA
MCCC 1K01826	*A. macleodii*	Western Pacific Ocean	Subsurface (100 m)	+	+	+	−	NA

Supplementary Table S2. *Cont.)*

Strain	Taxonomy	Origin	Depth	R9Y1	R9Y2	R9Y3	R8W[a]	R7M[b]
A14	*A. macleodii*	South China Sea	Subsurface (75 m)	+	+	+	−	NA
MCCC 1K00767	*A. macleodii*	Eastern Pacific Ocean	Mesopelagic (500 m)	+	+	+	−	NA
MCCC 1K01276	*A. macleodii*	Western Pacific Ocean	Mesopelagic (300 m)	+	+	+	−	NA
MCCC 1K00800	*A. macleodii*	Eastern Pacific Ocean	Bathypelagic (1000 m)	+	+	+	−	NA
MCCC 1A02046	*A. macleodii*	Indian Ocean	Bathypelagic (2391 m)	+	+	+	−	NA
MCCC 1K02456	*A. macleodii*	Northwestern Indian Ocean	Sediment (1818 m)	+	+	+	−	NA
MCCC 1K02451	*A. macleodii*	Northwestern Indian Ocean	Sediment (2009 m)	+	+	+	−	NA
MCCC 1K02444	*A. macleodii*	Northwestern Indian Ocean	Sediment (2540 m)	+	+	+	−	NA
MCCC 1K01703	*A. macleodii*	Atlantic Ocean	Sediment (2781m)	+	+	+	−	NA
MCCC 1K01716	*A. macleodii*	Atlantic Ocean	Sediment (2781 m)	+	+	+	−	NA
MCCC 1K02452	*A. macleodii*	Northwestern Indian Ocean	Olivine (3042 m)	+	+	+	−	NA
DSM26505	*A. macleodii*	Andaman Sea	N/A	+	+	+	−	NA
AD037	*A. macleodii*	Port Dickson, Malaysia	Surface	−	+	+	+	NA
MCCC 1K01358	*A. macleodii*	Eastern Pacific Ocean	Surface	−	+	+	−	NA
MCCC 1K01832	*A. macleodii*	Western Pacific Ocean	Subsurface (30 m)	−	−	−	+	NA
DSM26502	*A. macleodii*	Black Sea Karadag	Surface	−	−	−	−	NA
MCCC 1K01839	*A. macleodii*	Western Pacific Ocean	Surface	−	−	−	−	NA
MCCC 1K01823	*A. macleodii*	Western Pacific Ocean	Subsurface (75 m)	−	−	−	−	NA
A16	*A. macleodii*	South China Sea	Subsurface (75 m)	−	−	−	−	NA

Supplementary Table S2. *Cont.)*

Strain	Taxonomy	Origin	Depth	R9Y1	R9Y2	R9Y3	R8W[a]	R7M[b]
DSM26497	*A. macleodii*	Ionian Sea, Uranian Basin Western of Crete	Bathypelagic (3500 m)	–	–	–	–	NA
A25	*A. macleodii*	South China Sea	Bathypelagic (4219 m)	–	–	–	–	NA
A27	*A. macleodii*	South China Sea	Bathypelagic (4054 m)	–	–	–	–	NA
MCCC 1K01842	*A. macleodii*	Western Pacific Ocean	Subsurface (75 m)	NA	NA	NA	+	NA
MCCC 1F01223	*A. macleodii*	Xiamen, China	Algae culture	NA	NA	NA	+	NA
AD45	*A. macleodii*	Mediterranean Sea	Surface	NA	NA	NA	+	NA
MCCC 1K00562	*A. macleodii*	Eastern Pacific Ocean	Surface	+	+	+	NA	NA
MCCC 1K01333	*A. macleodii*	Pacific Ocean	Surface	+	+	+	NA	NA
MCCC 1K02752	*A. macleodii*	Atlantic Ocean	Surface	+	+	+	NA	NA
MCCC 1K02757	*A. macleodii*	Atlantic Ocean	Surface	+	+	+	NA	NA
A2	*A. macleodii*	Atlantic Ocean	Surface	+	+	+	NA	NA
A11	*A. macleodii*	South China Sea	Surface	+	+	+	NA	NA
A38	*A. macleodii*	South China Sea	Surface	+	+	+	NA	NA
MCCC 1A08375	*A. macleodii*	Eastern Pacific Ocean	Subsurface (25 m)	+	+	+	NA	NA
MCCC 1K01838	*A. macleodii*	Western Pacific Ocean	Subsurface (30 m)	+	+	+	NA	NA
MCCC 1K01203	*A. macleodii*	South China Sea	Subsurface (30 m)	+	+	+	NA	NA
MCCC 1K01646	*A. macleodii*	Atlantic Ocean	Subsurface (50 m)	+	+	+	NA	NA
MCCC 1K01647	*A. macleodii*	Atlantic Ocean	Subsurface (50 m)	+	+	+	NA	NA
MCCC 1K01648	*A. macleodii*	Atlantic Ocean	Subsurface (50 m)	+	+	+	NA	NA

Supplementary Table S2. *Cont.)*

Strain	Taxonomy	Origin	Depth	R9Y1	R9Y2	R9Y3	R8W[a]	R7M[b]
MCCC 1K01684	*A. macleodii*	Atlantic Ocean	Subsurface (50 m)	+	+	+	NA	NA
MCCC 1K01685	*A. macleodii*	Atlantic Ocean	Subsurface (50 m)	+	+	+	NA	NA
A6	*A. macleodii*	Philippine Sea	Subsurface (75 m)	+	+	+	NA	NA
A12	*A. macleodii*	South China Sea	Subsurface (75 m)	+	+	+	NA	NA
MCCC 1K01289	*A. macleodii*	Western Pacific Ocean	Mesopelagic (100 m)	+	+	+	NA	NA
MCCC 1K01109	*A. macleodii*	South China Sea	Mesopelagic (150 m)	+	+	+	NA	NA
A5	*A. macleodii*	Western Pacific Ocean	Mesopelagic (200 m)	+	+	+	NA	NA
A37	*A. macleodii*	South China Sea	Mesopelagic (200 m)	+	+	+	NA	NA
A39	*A. macleodii*	South China Sea	Mesopelagic (200 m)	+	+	+	NA	NA
MCCC 1K00282	*A. macleodii*	Eastern Pacific Ocean	Bathypelagic (1000 m)	+	+	+	NA	NA
MCCC 1K01191	*A. macleodii*	South China Sea	Bathypelagic (1000 m)	+	+	+	NA	NA
A20	*A. macleodii*	South China Sea	Bathypelagic (1000 m)	+	+	+	NA	NA
A24	*A. macleodii*	South China Sea	Bathypelagic (1000 m)	+	+	+	NA	NA
MCCC 1K01935	*A. macleodii*	South China Sea	Bathypelagic (1262 m)	+	+	+	NA	NA
MCCC 1K01957	*A. macleodii*	Western Pacific Ocean	Bathypelagic (1311 m)	+	+	+	NA	NA
MCCC 1K01949	*A. macleodii*	Western Pacific Ocean	Bathypelagic (1383 m)	+	+	+	NA	NA
MCCC 1K01725	*A. macleodii*	Atlantic Ocean	Sediment (1700 m)	+	+	+	NA	NA
MCCC 1K02450	*A. macleodii*	Northwestern Indian Ocean	Bathypelagic (2540 m)	+	+	+	NA	NA
MCCC 1K00283	*A. macleodii*	Eastern Pacific Ocean	Bathypelagic (3000 m)	+	+	+	NA	NA

Supplementary Table S2. *Cont.)*

Strain	Taxonomy	Origin	Depth	R9Y1	R9Y2	R9Y3	R8W[a]	R7M[b]
MCCC 1A09138	*A. macleodii*	Southern Atlantic Ocean	Bathypelagic (3399 m)	+	+	+	NA	NA
MCCC 1K00863	*A. macleodii*	Southern Pacific Ocean	Bathypelagic (3738 m)	+	+	+	NA	NA
A28	*A. macleodii*	South China Sea	Bathypelagic (3861 m)	+	+	+	NA	NA
A31	*A. macleodii*	South China Sea	Bathypelagic (3861 m)	+	+	+	NA	NA
MCCC 1K00848	*A. macleodii*	Southern Pacific Ocean	Bathypelagic (4258 m)	+	+	+	NA	NA
MCCC 1K00563	*A. macleodii*	Eastern Pacific Ocean	Bathypelagic (4860 m)	+	+	+	NA	NA
MCCC 1K00409	*A. macleodii*	Eastern Pacific Ocean	Bathypelagic (4898 m)	+	+	+	NA	NA
MCCC 1K02449	*A. macleodii*	Southwestern Indian Ocean	Bathypelagic (5152 m)	+	+	+	NA	NA
MCCC 1K01331	*A. macleodii*	Pacific Ocean	Bathypelagic (5302 m)	+	+	+	NA	NA
MCCC 1K00284	*A. macleodii*	Eastern Pacific Ocean	Bathypelagic (5089 m)	+	+	+	NA	NA
A13	*A. macleodii*	South China Sea	Subsurface (75 m)	−	+	+	NA	NA
A40	*A. macleodii*	South China Sea	Mesopelagic (200 m)	−	+	+	NA	NA
MCCC 1K00297	*A. macleodii*	Eastern Pacific Ocean	Mesopelagic (300 m)	−	+	+	NA	NA
MCCC 1K00564	*A. macleodii*	Pacific Ocean	Bathypelagic (5098 m)	−	+	+	NA	NA
MCCC 1K00097	*A. macleodii*	Northern Atlantic Ocean	Sediment (2577 m)	−	+	+	NA	NA
A4	*A. macleodii*	Pacific Ocean	Surface	−	−	−	NA	NA
A10	*A. macleodii*	South China Sea	Surface	−	−	−	NA	NA
A42	*A. macleodii*	Northern Pacific Ocean	Surface	−	−	−	NA	NA
MCCC 1K00561	*A. macleodii*	Eastern Pacific Ocean	Surface	−	−	−	NA	NA

Supplementary Table S2. *Cont.)*

Strain	Taxonomy	Origin	Depth	R9Y1	R9Y2	R9Y3	R8W[a]	R7M[b]
MCCC 1K01132	*A. macleodii*	South China Sea	Subsurface (30 m)	−	−	−	NA	NA
MCCC 1A09719	*A. macleodii*	Southern Atlantic Ocean	Subsurface (50 m)	−	−	−	NA	NA
A15	*A. macleodii*	South China Sea	Subsurface (75 m)	−	−	−	NA	NA
MCCC 1K00753	*A. macleodii*	South China Sea	Mesopelagic (450 m)	−	−	−	NA	NA
MCCC 1A03444	*A. macleodii*	South China Sea	Mesopelagic (812 m)	−	−	−	NA	NA
MCCC 1K00759	*A. macleodii*	South China Sea	Bathypelagic (1000 m)	−	−	−	NA	NA
A21	*A. macleodii*	South China Sea	Mesopelagic (100 m)	−	−	−	NA	NA
A33	*A. macleodii*	South China Sea	Mesopelagic (200 m)	−	−	−	NA	NA
A35	*A. macleodii*	South China Sea	Mesopelagic (200 m)	−	−	−	NA	NA
A41	*A. macleodii*	South China Sea	Mesopelagic (200 m)	−	−	−	NA	NA
A18	*A. macleodii*	South China Sea	Bathypelagic (1000 m)	−	−	−	NA	NA
A19	*A. macleodii*	South China Sea	Bathypelagic (1000 m)	−	−	−	NA	NA
A23	*A. macleodii*	South China Sea	Bathypelagic (1000 m)	−	−	−	NA	NA
MCCC 1K01190	*A. macleodii*	South China Sea	Bathypelagic (1000 m)	−	−	−	NA	NA
MCCC 1K02216	*A. macleodii*	Western Pacific Ocean	Bathypelagic (1200 m)	−	−	−	NA	NA
MCCC 1K01923	*A. macleodii*	South China Sea	Bathypelagic (1262 m)	−	−	−	NA	NA
MCCC 1K02267	*A. macleodii*	Western Pacific Ocean	Bathypelagic (1267 m)	−	−	−	NA	NA
MCCC 1A01084	*A. macleodii*	Indian Ocean	Bathypelagic (2488 m)	−	−	−	NA	NA
A32	*A. macleodii*	South China Sea	Bathypelagic (2739 m)	−	−	−	NA	NA

Supplementary Table S2. *Cont.)*

Strain	Taxonomy	Origin	Depth	R9Y1	R9Y2	R9Y3	R8W[a]	R7M[b]
A29	*A. macleodii*	South China Sea	Bathypelagic (3861 m)	−	−	−	NA	NA
A26	*A. macleodii*	South China Sea	Bathypelagic (4054 m)	−	−	−	NA	NA
MCCC 1A09498	*A. macleodii*	Southern Atlantic Ocean	Sediment (1598 m)	−	−	−	NA	NA
MCCC 1A06665	*A. macleodii*	Bali Beach, Indonesia	Beach	−	−	−	NA	NA
MCCC 1K00565	*A. abrolhosensis*	Eastern Pacific Ocean	Bathypelagic (5098 m)	+	+	+	+	NA
MCCC 1A09157	*A. abrolhosensis*	Southern Atlantic Ocean	Sediment	+	+	+	+	NA
MCCC 1A09130	*A. abrolhosensis*	Southern Atlantic Ocean	Sediment	+	+	+	+	NA
A22	*A. abrolhosensis*	South China Sea	Bathypelagic (1003 m)	+	+	+	NA	NA
MCCC 1K01033	*A. abrolhosensis*	Southern Pacific Ocean	Bathypelagic (5306 m)	+	+	+	NA	NA
MCCC 1F01092	*A. abrolhosensis*	Chiu-lung River	Sediment (0 m)	+	+	+	NA	NA
MCCC 1K01723	*A. abrolhosensis*	Atlantic Ocean	Sediment (1700 m)	+	+	+	NA	NA
MCCC 1K01726	*A. abrolhosensis*	Atlantic Ocean	Sediment (1700 m)	+	+	+	NA	NA
MCCC 1A09116	*A. abrolhosensis*	Southern Atlantic Ocean	Sediment (2596 m)	+	+	+	NA	NA
MCCC 1K01719	*A. abrolhosensis*	Atlantic Ocean	Sediment (2727 m)	+	+	+	NA	NA
MCCC 1K01720	*A. abrolhosensis*	Atlantic Ocean	Sediment (2727 m)	+	+	+	NA	NA
MCCC 1K01717	*A. abrolhosensis*	Atlantic Ocean	Sediment (2781 m)	+	+	+	NA	NA
MCCC 1K01724	*A. abrolhosensis*	Atlantic Ocean	Sediment (3110 m)	+	+	+	NA	NA
MCCC 1K01727	*A. abrolhosensis*	Atlantic Ocean	Sediment (3110 m)	+	+	+	NA	NA
MCCC 1A08167	*A. abrolhosensis*	Southern Atlantic Ocean	Seafloor rocks (2779 m)	+	+	+	NA	NA

Supplementary Table S2. *Cont.)*

Strain	Taxonomy	Origin	Depth	R9Y1	R9Y2	R9Y3	R8W[a]	R7M[b]
MCCC 1K00364	*A. abrolhosensis*	Eastern Pacific Ocean	Sediment (5368 m)	−	−	−	NA	NA
EC615	*A. mediterranea*	English Channel	Surface	+	+	+	+	NA
DE1	*A. mediterranea*	Adriatic Sea, Urania Basin	Bathypelagic (1000 m)	+	+	+	+	NA
UM7	*A. mediterranea*	Ionian Sea, Uranian Basin Western of Crete	Bathypelagic (3475 m)	+	+	+	+	NA
UM8	*A. mediterranea*	Ionian Sea, Uranian Basin Western of Crete	Bathypelagic (3475 m)	+	+	+	+	NA
UM4b	*A. mediterranea*	Ionian Sea, Uranian Basin Western of Crete	Bathypelagic (3455 m)	+	+	+	−	NA
MCCC 1A07988	*A. mediterranea*	Southern Atlantic Ocean	Bathypelagic (5610 m)	+	+	+	−	NA
U4	*A. mediterranea*	Ionian Sea, Uranian Basin Western of Crete	Bathypelagic (3475 m)	+	+	−	−	NA
U7	*A. mediterranea*	Ionian Sea, Uranian Basin Western of Crete	Bathypelagic (3500 m)	+	+	−	−	NA
U8	*A. mediterranea*	Ionian Sea, Uranian Basin Western of Crete	Bathypelagic (3500 m)	+	+	−	−	NA
MED64	*A. mediterranea*	Aegean Sea, Mediterranean	Surface	−	−	−	−	NA
MCCC 1A08050	*A. mediterranea*	Southern Atlantic Ocean	Sediment (2481 m)	−	−	−	−	NA
MCCC 1A05262	*A. mediterranea*	Southwestern Pacific Ocean	Bathypelagic (2695 m)	−	−	−	NA	NA
MCCC 1A08002	*A. mediterranea*	Southern Atlantic Ocean	Sediment (5610 m)	−	−	−	NA	NA
MCCC 1A09642	*A. mediterranea*	Southern Atlantic Ocean	Bathypelagic (1000 m)	+	+	+	NA	NA
MCCC 1F01155	*A. mediterranea*	South China Sea	Surface	+	+	+	NA	NA
MCCC 1F01157	*A. mediterranea*	South China Sea	Surface	+	+	+	NA	NA
MCCC 1K02027	*A. mediterranea*	South China Sea	Bathypelagic (1700 m)	+	+	+	NA	NA
MCCC 1K02248	*A. mediterranea*	Western Pacific Ocean	Bathypelagic (1100 m)	−	−	−	NA	NA

Supplementary Table S2. *Cont.)*

Strain	Taxonomy	Origin	Depth	R9Y1	R9Y2	R9Y3	R8W[a]	R7M[b]
A36	*A. australica*	South China Sea	Mesopelagic (200 m)	−	−	−	NA	NA
MCCC 1K02087	*Alteromonas sp.*	South China Sea	Bathypelagic (1700 m)	+	+	+	+	NA
MCCC 1K01057	*Alteromonas sp.*	South China Sea	Mesopelagic (700 m)	+	+	+	NA	NA
MCCC 1K01055	*Alteromonas sp.*	South China Sea	Surface	−	−	−	NA	NA

NA indicates that the data are not available.

[a]The host range data were obtained from Feng *et al*. (2021). Gray shadows indicate the same strain collection as R8W in Feng *et al.* (2021).

[b]The host range data were obtained from Ma *et al*. (2021).

Supplementary Table S3. Abundance and location information of R9Y-phages found through read mapping.

Run_accession	RPKM	Coverage (%)	sampling station	Latitude	Longitude	Depth (m)	Environment Feature
ERR599341	10.0222	83.8609	TARA_122	S 8° 59'49.08"	W 139° 11'48.48"	600	(MES) mesopelagic zone
ERR599345	5.1526	81.0629	TARA_138	N 6° 19'59.52"	W 102° 56'35.58"	60	(DCM) deep chlorophyll maximum layer
ERR599365	37.7394	88.6539	TARA_138	N 6° 19'59.52"	W 102° 56'35.58"	450	(MES) mesopelagic zone
ERR599366	8.3115	81.8022	TARA_137	N 14° 12'7.92"	W 116° 37'44.64"	375	(MES) mesopelagic zone

References

1. Feng X, Yan W, Wang A, Ma R, Chen X, Lin T-H, Chen Y-L, Wei S, Jin T, Jiao N, Zhang R. 2021. A novel broad host range phage infecting *Alteromonas*. Viruses 13:987.

2. Garcia-Heredia I, Rodriguez-Valera F, Martin-Cuadrado A-B. 2013. Novel group of podovirus infecting the marine bacterium *Alteromonas macleodii*. Bacteriophage 3:e24766.

3. Gao Y, Liu Q, Wang M, Zhao G, Jiang Y, Malin G, Gong Z, Meng X, Liu Z, Lin T. 2017. Characterization and genome sequence of marine *Alteromonas gracilis* phage PB15 isolated from the Yellow Sea, China. Current microbiology 74:821-826.

4. Kallies R, Kiesel B, Zopfi J, Wick LY, Chatzinotas A. 2017. Complete genome sequence of *Alteromonas* virus vB_AspP-H4/4. Genome announcements 5:e00914-17.

5. Wang D, Jiang Y, Xiao S, Wang M, Liu Q, Huang L, Xue C, Wang Q, Lin T, Shao H. 2019. Characterization and genome analysis of a novel *Alteromonas* phage JH01 isolated from the Qingdao coast of China. Current microbiology 76:1256-1263.

6. Gonzalez-Serrano R, Dunne M, Rosselli R, Martin-Cuadrado A-B, Grosboillot V, Zinsli LV, Roda-Garcia JJ, Loessner MJ, Rodriguez-Valera F. 2020. *Alteromonas* Myovirus V22 represents a new genus of marine bacteriophages requiring a tail fiber chaperone for host recognition. mSystems 5:e00217-20.

7. Zhang X, Liu Y, Wang M, Wang M, Jiang T, Sun J, Gao C, Jiang Y, Guo C, Shao H. 2020. Characterization and genome analysis of a novel marine *Alteromonas* phage P24. Current microbiology 77:2813-2820.

8. Ma R, Lai J, Chen X, Wang L, Yang Y, Wei S, Jiao N, Zhang R. 2021. A novel phage infecting *Alteromonas* represents a distinct group of siphophages infecting diverse aquatic copiotrophs. mSphere 6:e00454-21.

9. Wang Z, Zhang F, Liang Y, Zheng K, Gu C, Zhang W, Liu Y, Zhang X, Shao H, Jiang Y. 2021. Genome and ecology of a novel *Alteromonas* podovirus, ZP6, representing a new viral genus, *Mareflavirus*. Microbiology spectrum 9:e00463-21.

10. Lopes A, Tavares P, Petit M-A, Guérois R, Zinn-Justin S. 2014. Automated classification of tailed bacteriophages according to their neck organization. BMC genomics 15:1-17.
